# Medical History from the Earliest Times

**Published:** 1892-04-30

**Authors:** E. T. Withington


### Medical History from the Earliest Times.

By E. T. Withington, M.A., M.B. Oxon.

### VI.—HINDOO MEDICINE.

The earliest documents of that Indo-Germanic race to which we ourselves belong are the Vedas, or books of revealed "wisdom"—the two words having the same root. The oldest of these, the Rig-Veda (knowledge of praise), consisting of hymns composed B C. 2000—1000, already mentions a special class of physicians; it also contains passages in praise of the healing power of herbs, and waters, and notices at least two diseases—phthisis and leprosy. But the fourth, or Atharva-Veda (knowledge of spells), compiled about 700 B.C., is, as might be expected, the most medical in character of the four great religious books. As we have already considered this aspect of the healing art, only one example need be given here; an invocation against Takman, the demon of fever, evidently adapted to high caste patients only. "May refusal meet Takman, who has glowing weapons. O Takman, go to the Mujavant or further. Attack the Sudra (low caste) woman, the teeming one; shake her, O, Takman." The most select remedies seem to have been preserved for princes; thus, when the son of Bimbisara—king of Magadha, about 600 B.C.—fainted, he was placed in six tubs full of fresh butter, and afterwards in a seventh filled with the most costly sandal wood, after which it is interesting to know that the prince survived, and succeeded his father.

Though the incantations of the Atharva-Veda were, doubtless, recited by Brahmans, it is important to notice that the physicians of the Vedic age did not belong to this priestly caste. They are classed in the ancient laws of Manu among those unclean persons who are excluded from the funeral feasts, and their origin is attributed in the Brahmanic writings to intermarriages between men and women of different caste. The majority, however, probably belonged to the great Hindoo middle-class, the Vaisyas (agriculturists and traders), and in later times a physician might take as pupil a member of any caste except a Sudra.

Besides the four great religious Vedas, the Hindoos have certain supplementary "Upa"-Vedas later in date, and dealing with more worldly subjects—medicine, music, architecture, &c. The first of these is the Ayur-Veda (knowledge of life), a term not confined to any particular book, but including all works on medicine thought to have been in any way supernaturally "revealed." It is especially applied to the works of Charaka, said to have been revealed by Indra himself through the medium of a Rishi, or sage, and to those of Susruta, dictated by the divine physician Dhanwantari, who became incarnate for that purpose.

The story of the birth of this Hindoo Æsculapius is too curious to be omitted. A blight had fallen upon the universe, and the anxious gods came to their father, Vishnu, for advice. He declared that they must obtain the "Amrita," or drink of immortality, and that for this purpose the ocean of milk must be churned. Gods and demons, forgetting for a time their hostility, united in this stupendous work. The great serpent, Vasuki, twined himself round the mountain Mandara, and the gods and demons grasping the monster's tail, swung the mountain round in the milk ocean upon the back of Vishnu himself, who lay in the shape of a huge tortoise at the bottom. Long they laboured, and the demons, who were nearest the serpent's head, became permanently blackened by the poisonous fumes from his hood; but at last the work was done, and there rose from the churned ocean the moon, a marvellous tree, and sacred cow, the goddesses of love, wine, and beauty, and, finally, the white-robed physician, Dhanwantari, with the cup of the Amrita in his hand. In pity for the ills of mortals, he caused himself to be born on earth as a prince of Benares, and having retired to the woods as a hermit, after the manner of ancient Hindoo princes, dictated to Susruta, a son of the famous warrior-sage Visvamitra, his Ayur-Veda.

In the works of Charaka and Susruta we find a condition of medical and surgical knowledge not unworthy to be compared with that of the Hippocratic writers, of which, however, there is no indication in the earlier Vedic age, and which had left no traces of its existence in the sixteenth century of our era. Only a few particulars can here be given. The most striking feature is the high place assigned to surgery, a fact sufficient in itself to disprove the priestly origin of these works. "Surgery," says Susruta, "is the first and highest division of the healing art, least liable to fallacy, pure in itself, perpetual in its applicability, the worthy produce of heaven, the sure source of fame on earth." At the same time he emphasises the unity of medicine: "He who only knows one branch of his art is like a bird with one wing." Practical and theoretic knowledge must be combined: "He who is versed only in books will be alarmed and confused, like a coward on the battle-field, when in face of active disease; he who rashly engages in practice without previous study of written science is entitled to no respect from mankind, and merits punishment from the king; but he who combines reading with experience proceeds safely and surely like a chariot on two wheels." He similarly warns his pupils against unintelligent repetition from books: the student who thus obtains his knowledge "is like an ass with a burden of sandal-wood, for he knoweth the weight but not the value thereof."

The most famous achievement of Hindoo surgery is the manufacture of new noses by flaps taken from the cheeks or forehead, an operation specially demanded in a land where despotic rulers and jealous husbands were singularly additec to mutilating their victims. But Susruta also mentions the division of the supra-orbital nerve for neuralgia, and laparotomy and suture of the intestine for obstruction or injury—operations lately re-introduced into medicine. He describes more than one hundred instruments, the first and best of which is the hand, and carefully distinguishes twelve species of leeches, a section of his work afterwards highly valued by the Arabs, who in the eighth century A.D. translated portions of the works of both the great Hindoo physicians.

And this brings us to the important question, When did this remarkable development of Hindoo medicine, which arose and vanished so mysteriously, actually take place? The lower limit is fixed by the above-mentioned translations at about 750 A.D., but it is very difficult to find a higher one. Older writers, relying on the fact that both Susruta and Charaka are mentioned in the great epic, Mahabharata, considered that the works attributed to them must be at least as old as the Homeric poems. But the Mahabharata underwent revisions and additions to a very late date, and recent Sanscrit scholars assert that the language of the medical books indicates an age certainly not long anterior to Alexander's invasion, B.C. 327, and possibly even later than this. Now these dates, B.C. 327—A.D. 750, nearly correspond to the period of the predominance of Buddhism in India, and it seems highly probable that this Buddhist millennium was also the golden age of Hindoo medicine. The brotherly love and sympathetic pity inculcated by the "Enlightened" were more likely to favour the progress of the holiest of arts than the caste prejudices and endless formalities of Brahmanism; nor are we without definite indication of the high honour in which Buddhists held the physician. Thus one of the chief evils of poverty is that the poor man cannot get a physician or medicine, and travellers are warned against settling in a land where there are not five things—a king, a river, rich men, teachers, and—physicians. When King Asoka, the Constantine of Buddhism, made that faith the religion of his empire, B.C. 250, he issued a series of edicts, two of which have reference to our present subject. In the first, "the man of loving spirit, beloved of the gods," declares that no animal is to be put to death, and we may, perhaps, connect this with the extraordinary passage in Susruta which forbids medical aid to hunters and to all who kill or trap animals. In the second, Asoka says that he has established two "cures," one for men and another for animals, not only throughout his own dominions, but in those of neighbouring monarchs, and has arranged for the collection and planting of medicinal herbs in those places where they do not exist. We cannot here discuss the precise nature of these "cures," and to what extent they may have resembled hospitals, or even medical schools, but the passage is enough to show the great attention and encouragement given by Buddhist rulers to the healing art.

In conclusion, the following anecdote from a Buddhist writer indicates the richness of the Hindoo materia medica, a subject which may yet repay investigation. Jivaka, afterwards physician to Buddha himself, having completed his medical education, was sent by his master, as a final test, into the forest, with orders to bring back specimens of those herbs which were of *no use* in medicine. After long wandering in the jungle, the youth returned empty-handed; he could find no plant that had not some medicinal virtues. The master, of course, declared that to such a pupil there was nothing more to be taught.

